# Effectiveness of remote risk-based monitoring and potential benefits for combination with direct data capture

**DOI:** 10.1186/s13063-024-08242-2

**Published:** 2024-06-14

**Authors:** Osamu Yamada, Shih-Wei Chiu, Toru Nakazawa, Satoru Tsuda, Mitsuhide Yoshida, Toshifumi Asano, Taiki Kokubun, Kazuki Hashimoto, Munenori Takata, Suzuka Ikeda, Yosuke Kawabe, Yuko Tamura, Takuhiro Yamaguchi

**Affiliations:** 1 Division of Biostatistics, Graduate School of Medicine, Tohoku University, 1-1 Seiryo-Machi, Aoba-Ku, Sendai, Miyagi 980-8574 Japan; 2 Clinical Research Data Center, Tohoku University Hospital, 1-1 Seiryo-Machi, Aoba-Ku, Sendai, Miyagi 980-8574 Japan; 3 Department of Ophthalmology, Graduate School of Medicine, Tohoku University, 1-1 Seiryo-Machi, Aoba-Ku, Sendai, Miyagi 980-8574 Japan; 4 Department of Ophthalmic Imaging and Information Analytics, Graduate School of Medicine, Tohoku University, 1-1 Seiryo-Machi, Aoba-Ku, Sendai, Miyagi 980-8574 Japan; 5 Department of Retinal Disease Control, Graduate School of Medicine, Tohoku University, 1-1 Seiryo-Machi, Aoba-Ku, Sendai, Miyagi 980-8574 Japan; 6 Department of Advanced Ophthalmic Medicine, Graduate School of Medicine, Tohoku University, 1-1 Seiryo-Machi, Aoba-Ku, Sendai, Miyagi 980-8574 Japan; 7 Department of Collaborative Program for Ophthalmic Drug Discovery, Graduate School of Medicine, Tohoku University, 1-1 Seiryo-Machi, Aoba-Ku, Sendai, Miyagi 980-8574 Japan; 8 beagle Co., Ltd, 1-1-25 Toranomon, Chuo-Ku, Tokyo, 105-0001 Japan

**Keywords:** Prospective study, Remote monitoring, Risk-based monitoring, Clinical trial monitoring, Direct data capture, eSource

## Abstract

**Background:**

In recent years, alternative monitoring approaches, such as risk-based and remote monitoring techniques, have been recommended instead of traditional on-site monitoring to achieve more efficient monitoring. Remote risk-based monitoring (R2BM) is a monitoring technique that combines risk-based and remote monitoring and focuses on the detection of critical data and process errors. Direct data capture (DDC), which directly collects electronic source data, can facilitate R2BM by minimizing the extent of source documents that must be reviewed and reducing the additional workload on R2BM. In this study, we evaluated the effectiveness of R2BM and the synergistic effect of combining R2BM with DDC.

**Methods:**

R2BM was prospectively conducted with eight participants in a randomized clinical trial using a remote monitoring system that uploaded photographs of source documents to a cloud location. Critical data and processes were verified by R2BM, and later, all were confirmed by on-site monitoring to evaluate the ability of R2BM to detect critical data and process errors and the workload of uploading photographs for clinical trial staff. In addition, the reduction of the number of uploaded photographs was evaluated by assuming that the DDC was introduced for data collection.

**Results:**

Of the 4645 data points, 20.9% (*n* = 973, 95% confidence interval = 19.8–22.2) were identified as critical. All critical data errors corresponding to 5.4% (*n* = 53/973, 95% confidence interval = 4.1–7.1) of the critical data and critical process errors were detectable by R2BM. The mean number of uploaded photographs and the mean time to upload them per visit per participant were 34.4 ± 11.9 and 26.5 ± 11.8 min (mean ± standard deviation), respectively. When assuming that DDC was introduced for data collection, 45.0% (95% confidence interval = 42.2–47.9) of uploaded photographs for R2BM were reduced.

**Conclusions:**

R2BM can detect 100% of the critical data and process errors without on-site monitoring. Combining R2BM with DDC reduces the workload of R2BM and further improves its efficiency.

**Supplementary Information:**

The online version contains supplementary material available at 10.1186/s13063-024-08242-2.

## Background

The cost of conducting clinical trials has been increasing as they become larger and more complex [1]. This can be partially attributed to the traditional monitoring approach that ensures data reliability via intensive on-site visits and 100% source data verification (SDV), regardless of compound characteristics or clinical trial designs. Despite considerable monetary investment for traditional monitoring, 100% SDV has a negligible effect on data quality, as only a small percentage of case report form (CRF) data is corrected by SDV, and most data errors do not impact the interpretation of the clinical trial results [2–4].

In 2013, American, European, and Japanese regulatory authorities issued updated guidelines on the risk-based monitoring (RBM) approach to achieve more efficient monitoring. These guidelines recommend identifying critical data and processes and shifting monitoring activities from excessive reliance on SDV to prevent and mitigate important and likely risks [5–7]. RBM has become widely known in these updated guidelines; however, it has not yet been fully introduced into clinical trials because of the lack of evidence of its effectiveness and familiarity with appropriate RBM approaches [8].

Remote monitoring also ensures data integrity as an alternative to traditional on-site monitoring, reducing travel time and costs [9–11]. Remote monitoring has received considerable attention owing to the coronavirus disease 2019 (COVID-19) pandemic [8]; however, it requires a site-specific infrastructure for remote access to electronic health records (EHR), or it generates additional workloads where the clinical trial staff are required to scan or capture photographs of source documents [12, 13]. These disadvantages make the clinical trial staff reluctant to support remote monitoring approaches.

Direct data capture (DDC) is a method used to directly enter clinical trial data as electronic source (eSource) data into an electronic CRF (eCRF) unlike electronic data capture (EDC) that requires transcriptions from source documents to eCRF [14, 15]. DDC can eliminate unnecessary data duplication in source documents, reduce the possibility of transcription errors from source documents to eCRF, and promote real-time access to clinical trial data without reviewing source documents [14–16]. This results in minimizing the number of source documents that must be reviewed at clinical trial sites or reducing the additional workload of capturing source documents for remote monitoring. However, source data that have already been recorded in EHR before a clinical trial started or were generated through the EHR system, such as medical history, prescription records, and results of clinical laboratory tests measured in a clinical trial site, still need transcriptions even when DDC is introduced for data collection. Therefore, the amount of clinical trial data that can be collected as eSource data by the DDC and its effects on the workload of remote monitoring remain unelucidated.

Previously, our retrospective study revealed that remote risk-based monitoring (R2BM), a monitoring technique that combines RBM and remote monitoring, detected both critical data and process errors, thereby saving travel time and costs for traditional on-site monitoring [17]. In this study, we prospectively evaluated the effectiveness of R2BM in a clinical trial to confirm the reproducibility of the results. In addition, to evaluate the impact of introducing DDC on the effectiveness of R2BM, we analyzed the amount of data that can be collected as eSource data without transcriptions from source documents and the effects on the workload of remote monitoring by assuming that DDC was introduced for data collection instead of EDC in a clinical trial.

## Methods

### Characteristics and participants of the selected clinical trial

A prospective, double-blind, randomized controlled trial of central retinal artery occlusion (trial ID in the Japan Registry of Clinical Trials: jRCT2021190013) was selected to evaluate the R2BM methodology. The study duration for each participant was 12 weeks and included 6 visits: weeks 0, 1, 2, 4, 8, and 12. Twenty participants were randomized and administered the study treatment. R2BM was conducted with only the last 8 of the 20 participants because the present study started in the middle of the selected clinical trial. On-site monitoring reviewing all records in the source documents of the 20 participants was conducted within 4 weeks after weeks 4 and 12 for each participant by on-site monitoring clinical research associates (on-site CRAs). EDC was used for data collection, and data entered into the EDC were reviewed on time by a data manager (DM) without confirming any source document.

### Remote monitoring system

A remote monitoring system called beagle View^®^, developed by beagle Co. Ltd., was used for R2BM. The system is a cloud-based application downloaded to a tablet device prepared exclusively for this study that enables remote monitoring through the following steps: (1) clinical trial staff capture photographs of source documents, such as EHR screens and paper worksheets, using the device’s camera; (2) clinical trial staff upload the photographs immediately to a folder designated for each participant’s visit to a secure cloud location by selecting several photographs saved on the tablet device without any additional procedure such as changing the file name; and (3) a remote monitoring CRA (remote CRA), different from on-site CRAs, views the photographs remotely in a private room.

Personal information was included in the uploaded photographs without masking. Therefore, appropriate written informed consent was obtained from all participants for handling personal information. In addition, all researchers received training on procedures to protect the participants’ personal information.

### R2BM plan

Data and processes related to the following items were defined as critical: (1) informed consent, (2) eligibility criteria, (3) visit date, (4) randomization, (5) study treatments, (6) primary endpoint, (7) treatment compliance, (8) stratification factors for the primary endpoint, (9) adverse events, and (10) discontinuation. The risks that could lead to critical process errors were identified by referring to the risk assessment and categorization tool developed by TransCelerate BioPharma Inc. [18].

To ensure the capture of photographs of source documents that the remote CRA needed to review, a source document identification list defining when and which source documents should be captured and uploaded was created by discussing with the clinical trial staff. Only the source documents necessary to confirm 100% of the critical data, critical processes, and identified risks were included in the list.

The R2BM plan required the clinical trial staff to capture and upload only photographs of the source documents defined in the list within 2 weeks after weeks 0, 1, 2, 4, and 12 for each participant. The photographs from week 8 were captured and uploaded simultaneously with those from week 12.

The remote CRA verified only critical data (targeted SDV) and reviewed only critical processes (targeted source data review; targeted SDR) without delay after uploading the photographs. If the remote CRA found the photograph inappropriate or missing, the clinical trial staff were asked to re-capture and upload the photographs.

### Implementation of R2BM

The remote CRA independently performed R2BM on the same eight participants in parallel with on-site monitoring by the on-site CRAs and data review by the DM. No data and process errors were shared between the remote CRA and the on-site CRAs or the DM.

After completing the R2BM, the remote CRA visited the clinical trial site and confirmed all data, processes, and source documents to evaluate the ability of R2BM to detect data and process errors and the accuracy of the uploaded photographs.

### Evaluation of R2BM methodology

#### Evaluation of the ability of R2BM to detect data and process errors

Data errors were defined as eCRF data corrected at least once. Process errors were defined as source records determined or else suspected to be protocol deviations. Because on-site monitoring, R2BM, and data review by DM were conducted independently and in parallel, we considered whether data and process errors detected by each method were also theoretically detectable by other methods based on the definitions in Table 1 and tallied.

**Table 1:** Definitions of data errors theoretically detectable by each method

Method	Definition
On-site monitoring	All data errors were regarded as theoretically detectable due to on-site CRAs accessing all source documents
R2BM	Only data errors in the uploaded photographs were regarded as theoretically detectable since the remote CRA could access only source documents uploaded to the cloud location
Data review by DM	Only data errors, such as typographical errors, contradiction in data between eCRF data, and data entered not following eCRF completion guidelines, were regarded as theoretically detectable since the DM could not access any source documents

*R2BM* remote risk-based monitoring, *DM* data manager, *CRA* clinical research associate, *eCRF* electronic case report form

#### Evaluation of the additional workload of R2BM for clinical trial staff

We measured the time to capture and upload photographs in units of 5 min and counted the number of uploaded photographs. Fractional minutes were rounded up to the closest 5-min unit.

#### Evaluation of uploaded photograph accuracy

Because R2BM was conducted based on information included in uploaded photographs, it was necessary to evaluate their accuracy. After completing the R2BM, the remote CRA visited the clinical trial site and confirmed all source documents. These documents were compared with those uploaded to the cloud, and the number of inappropriate photographs was counted and classified into the categories shown in Table 2.

**Table 2:** Definitions of photographs judged as inappropriate by the remote clinical research associate

Category	Definition
(A) Illegible photos	Photographs of insufficient image quality
(B) Incomplete photos	Photographs that did not include all required information
(C) Missed but detected photos	Photographs that should have been captured but were not and whose absence was detected during R2BM
(D) Missed and undetected photos	Photographs that should have been captured but were not and whose absence was not detected during R2BM
(E) Photos in incorrect folders of the correct participant	Photographs that were not uploaded to the designated folder, however in the other folders belonging to the correct participant
(F) Photos in incorrect participants’ folders	Photographs that were uploaded to folders belonging to other participants

*R2BM* remote risk-based monitoring

#### Evaluation of the period from each participant’s visit to photograph upload

Because clinical trial monitoring should be conducted in a timely manner following the monitoring plan, uploading photographs within 2 weeks after each participant’s visit was defined in the R2BM plan. To evaluate compliance with that requirement, the number of times to upload and the number of days from each participant’s visit to the upload of photographs were determined.

### Evaluation of the synergistic effects of DDC on R2BM methodology

By entering clinical trial data directly into eCRF, DDC can minimize the transcription of clinical trial data from source documents to the eCRF and reduce the number of data points requiring SDV. This may have reduced the number of photographs of source documents captured for R2BM. The following evaluations were conducted to clarify this benefit, assuming DDC was introduced for data collection instead of EDC in this study.

#### Evaluation of the number of data points that can be entered directly into eCRFs without transcriptions by introducing DDC

Not all data can be entered directly into the eCRFs without transcriptions, even though DDC is used for data collection because data generated in daily medical practice are recorded in the EHRs as source data. To evaluate how much data would be entered directly into the eCRFs without transcriptions by introducing DDC, the eCRF data were classified into category I (data not requiring transcriptions and SDV by introducing DDC) or category II (data requiring transcriptions and SDV despite introducing DDC) by discussing with the clinical trial staff and then counting. Detailed definitions of the categories are illustrated in Table 3.

**Table 3:** Definitions of data points classified as category I or category II

Category	Definition
Category I	The data were recorded only for the selected trial and could be directly entered into the eCRFs without ensuring consistency with other source data, e.g., data related to participant characteristics, vital signs, blood collection date and time, and ophthalmological tests recorded only for the selected clinical trial
Category II	The data were already recorded in EHR in daily medical practice or generated through the EHR system during the selected trial and could not be directly entered into the eCRFs, e.g., data on medical history, prescriptions for concomitant medications, and clinical laboratory tests measured at the selected clinical trial site

*EHR* electronic health record, *eCRFs* electronic case report forms

#### Evaluation of the proportion of data errors detectable only by SDV despite introducing DDC

Errors in the data classified as category I would not occur or could be detected by data review because the data can be entered directly into the eCRFs without transcriptions by introducing DDC. Although data classified as category II require SDV, errors in these data can be further classified into errors detectable by data review (category II-A) or only by SDV (category II-B). To evaluate how many data errors were detectable only by SDV when DDC was introduced (category II-B), the eCRF data errors were classified into one of the categories and counted.

#### Evaluation of the workload of R2BM reduced by introducing DDC

When DDC was introduced for data collection, data classified as category II would still require transcriptions and SDV. In the R2BM methodology focusing only on critical data, the clinical trial staff are only required to capture photographs of source documents, including critical data classified as category II. To evaluate how much the workload of R2BM was reduced by introducing DDC, the proportion of photographs not including critical data classified as category II was determined.

### Statistical analysis

Data points, the proportion of data errors, the accuracy of uploaded photographs, and the workload of R2BM that could be reduced by introducing DDC were calculated as proportions and presented as percentages and frequencies with 95% confidence intervals (CI) [19]. The number of uploaded photographs and the time to capture and upload photographs is expressed as mean ± standard deviation (SD). The number of days from each participant’s visit to the upload of photographs is shown as the median and interquartile range (IQR).

## Results

### Number and classification of data points

**Fig. 1:**
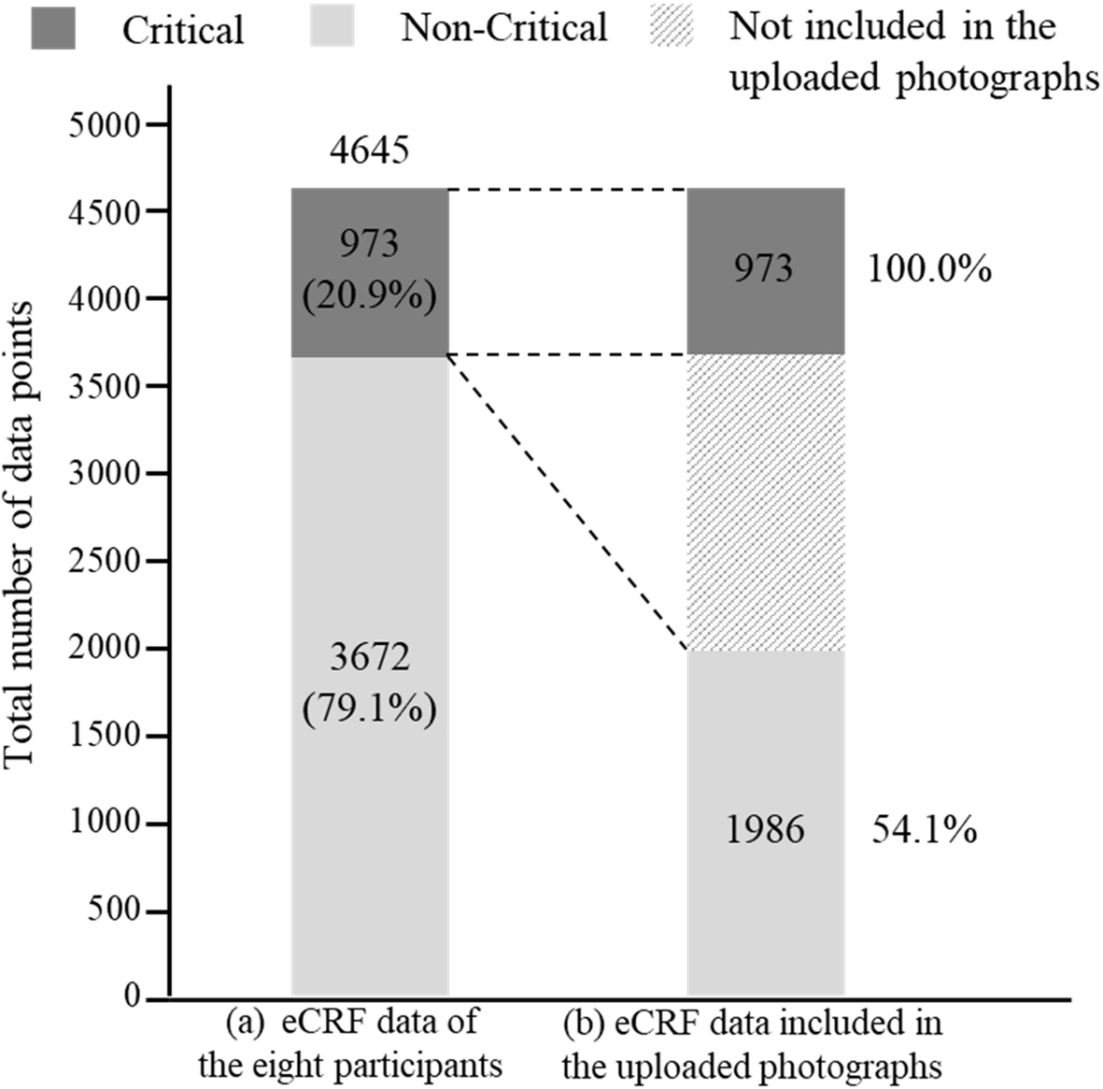
Total number of data points and proportion of critical and non-critical data. Critical: critical data; non-critical: non-critical data

The total number of data points entered in the CRFs of the eight participants was 4645, and the proportion of data points defined as critical was 20.9% (*n* = 973, 95% CI = 19.8–22.2), as shown in Fig. 1a. Of the total data points, 100% of the critical data points and 54.1% of the non-critical data points were included in the uploaded photographs, as shown in Fig. 1b.

### Evaluation of the R2BM methodology

#### The ability of R2BM to detect data and process errors

**Fig. 2:**
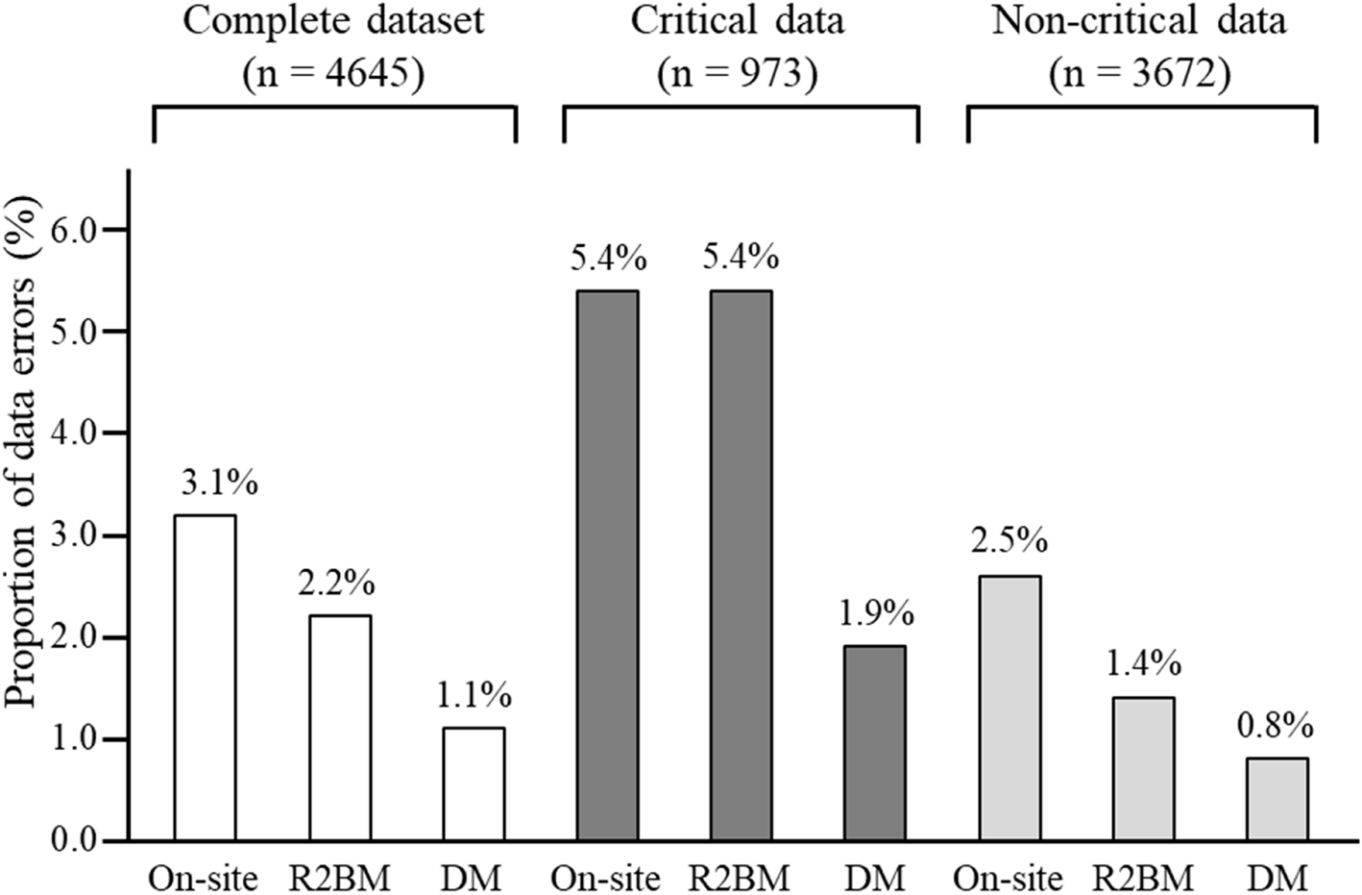
Proportion of data errors theoretically detectable by each method. On-site, on-site monitoring; R2BM, remote risk-based monitoring; DM, data review by the data manager

The proportions of data errors theoretically detectable by each method are shown in Fig. 2. In critical data, the proportions of data errors theoretically detectable by on-site monitoring, R2BM, and data review by DM were 5.4% (*n* = 53, 95% CI = 4.1–7.1), 5.4% (*n* = 53, 95% CI = 4.1–7.1), and 1.9% (*n* = 19, 95% CI = 1.2–3.0), respectively. All critical data errors could be detected by R2BM. For non-critical data, the difference in the proportion of data errors between on-site monitoring and R2BM was only 1.1% (2.5–1.4%). Two critical process errors and one non-critical process error occurred for eight participants, as shown in Table 4. All these were theoretically detectable by on-site monitoring and R2BM. The DM data review could not detect a critical process error related to eligibility confirmation.

**Table 4:** Number of process errors theoretically detectable by each method

		The number of process errors detected by each method		
		On-site	R2BM	DM
Critical process errors	Eligibility confirmation	1	1	0
	Prohibited concomitant medication	1	1	1
Non-critical process errors	Examinations related to other than the primary endpoint	1	1	1

On-*site* on-site monitoring, *R2BM* remote risk-based monitoring, *DM* data review by the data manager

#### The additional workload of R2BM for clinical trial staff

The total number of uploaded photographs was 1237, and the mean number of photographs per visit per participant was 34.4 ± 11.9 (mean ± SD), as shown in Additional file 1: Table S1(a). The total time to capture and upload photographs was 955 min, and the mean time per visit per participant was 26.5 ± 11.8 min (mean ± SD), as shown in Additional file 1: Table S1(b).

#### Uploaded photograph accuracy

The proportion of photographs that were not captured or uploaded appropriately is shown in Table 5. The proportion of photographs classified (A) to (F) was nominal. No illegible photos (*n* = 0, 95% CI = 0.0–0.3) and 0.1% (*n* = 1, 95% CI = 0.0–0.5) of incomplete photos, out of 1237, were observed. Of the source documents that should have been captured, 2.3% (*n* = 28, 95% CI = 1.5–3.4) was missed but detected during the R2BM, and only 0.1% (*n* = 1, 95% CI = 0.0–0.5) was missed and undetected during the R2BM. Of the 2.6% (*n* = 32, 95% CI = 1.8–3.6) photos in incorrect folders of the correct participant and 3.2% (*n* = 3, 95% CI = 2.3–4.3) photos in incorrect participants’ folders, all were detected by reviewing the contents of the uploaded photographs during the R2BM.

**Table 5:** Proportions of photographs that were not captured or uploaded appropriately

Category	Participant no								Total	Proportion
	01	02	03	04	05	06	07	08		
(A) Illegible photos										0.0% (0/1237)
(B) Incomplete photos				1					1	0.1 (1/1237)
(C) Missed but detected photos	1			3	1	8	3	12	28	2.3% (28/1237)
(D) Missed and undetected photos				1					1	0.1% (1/1237)
(E) Photos in incorrect folders of the correct participant	2			3	4			22	32	2.6% (32/1237)
(F) Photos in incorrect participants’ folders				11		5		23	39	3.2% (39/1237)

Definitions of category (A) to (F) are shown in Table 2

#### Period from each participant’s visit to photograph upload

The total number of visits by the 8 participants was 43, the number of times the photographs were uploaded was 12, and the median (IQR) number of days from each participant’s visit to the upload of photographs was 80 (52–113).

### Evaluation of the synergistic effects of DDC on R2BM methodology

#### Number of data points that can be entered directly into eCRFs without transcriptions by introducing DDC

**Fig. 3:**
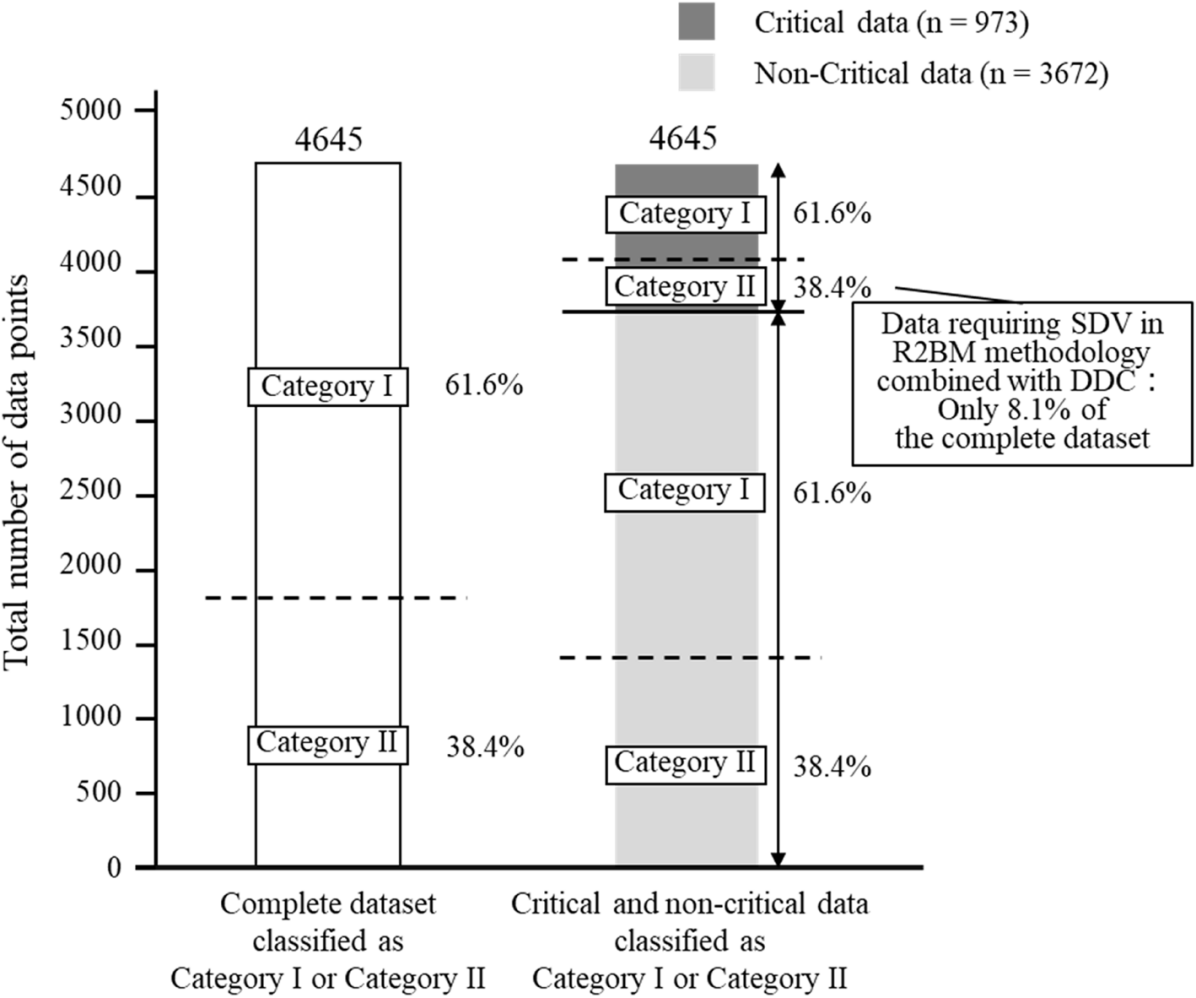
Number of data points classified as categories I or II. Category I: data not requiring transcriptions and source data verification by introducing direct data capture; category II: data requiring transcriptions and source data verification despite introducing direct data capture

The proportion of data points classified as category I was 61.6% (*n* = 2861, 95% CI = 60.2–63.0) in the complete dataset, 61.6% (*n* = 599, 95% CI = 58.4–64.6) in the critical data, and 61.6% (*n* = 2262, 95% CI = 60.0–63.2) in the non-critical data (Fig. 3). The proportion of data points classified as category II was 38.4% (*n* = 374, 95% CI = 35.4–41.6) in the critical data, equivalent to only 8.1% (*n* = 374, 95% CI = 7.3–8.9) of the complete dataset.

#### Proportion of data errors detectable only by SDV despite introducing DDC

**Fig. 4:**
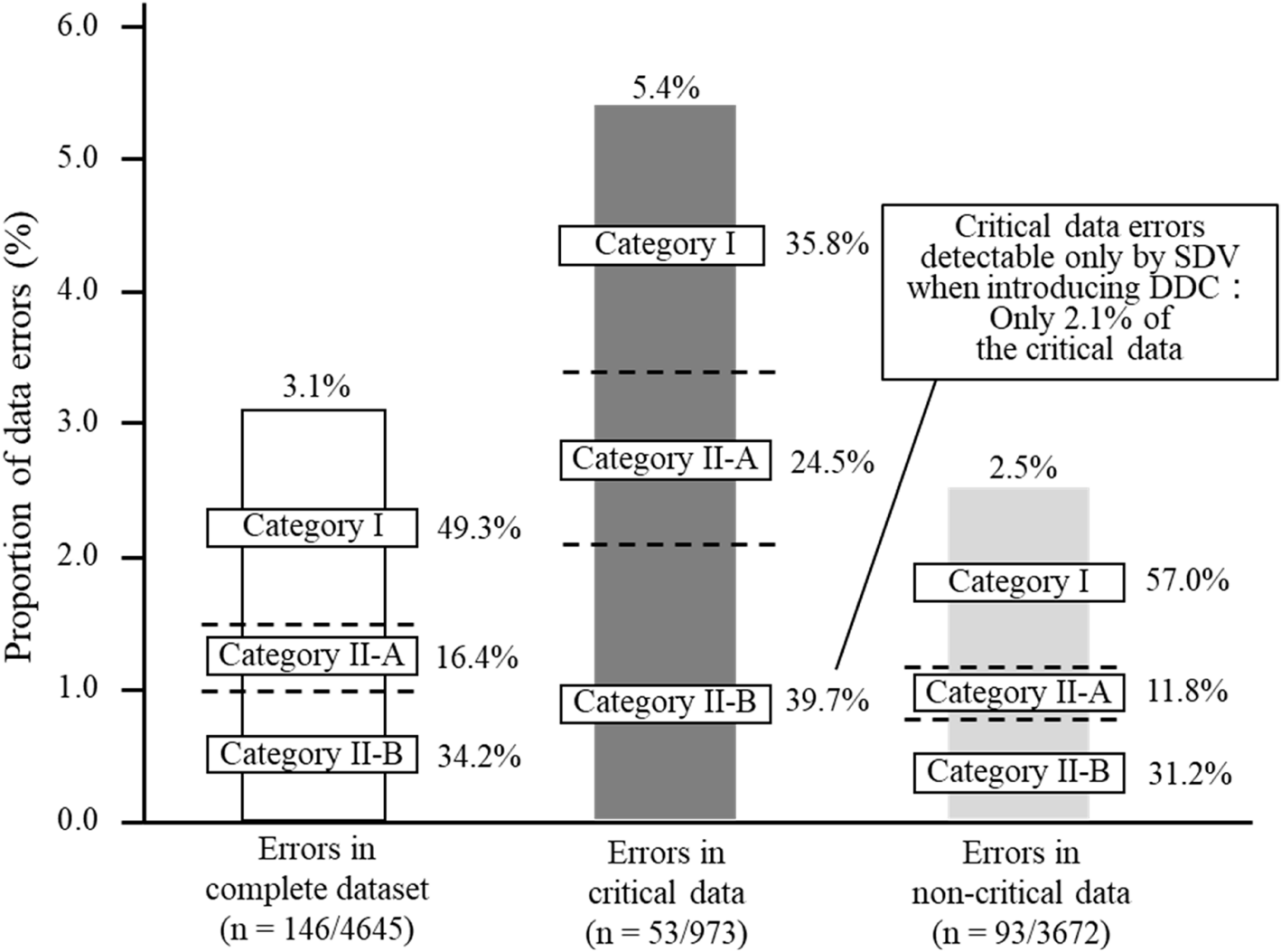
Proportion of data errors classified as category I, category II-A, or category II-B. Category I: data not requiring transcriptions and source data verification, with errors detectable by data review; category II-A: data requiring transcriptions and source data verification, with errors detectable by data review as well; category II-B: data requiring transcriptions and source data verification, with errors detectable only by source data verification

Of the data errors in the complete dataset, critical data, and non-critical data, 49.3% (*n* = 72, 95% CI = 41.0–57.7), 35.8% (*n* = 19, 95% CI = 23.1–50.2), and 57.0% (*n* = 53, 95% CI = 46.3–67.2) were regarded as data errors classified into category I, respectively (Fig. 4). Of the data errors in the critical data, 39.7% (*n* = 21, 95% CI = 26.5–54.0) were regarded as data errors detectable only by SDV (category II-B), which was equivalent to only 2.1% of the critical data.

#### Workload of R2BM reduced by introducing DDC

Of the 1237 uploaded photographs, 557 did not include critical data classified as category II (Additional file 1: Table S2). Of the total uploaded photographs, 45.0% (95% CI = 42.2–47.9) could be reduced by introducing DDC.

## Discussion

The primary purpose of this study was to prospectively evaluate whether R2BM was an effective monitoring method that ensured human subject protection and the reliability of trial results.

In this study, the proportion of data errors in the complete dataset was 3.1%, similar to that reported in other studies [2, 17, 20, 21]. Because data errors occurred in only a small percentages of the eCRF data, it was considered more efficient when SDV focused only on critical data, and resources for SDV were allocated to detect process errors and mitigate risks. This study and our previous study [17] showed that remote SDV detected 100% of the critical data errors, which is consistent with other studies on remote SDV [9, 10]. The difference in the proportion of noncritical data errors between on-site monitoring and R2BM was only 1.1%. As reported in other studies [3, 4], even if these non-critical data errors were overlooked or not intentionally confirmed, there would be no major impact on the interpretation of trial results. These findings indicate that remote SDV focusing only on critical data ensures data integrity in clinical trials. In addition, all critical process errors were detected by R2BM, indicating that it enabled remote SDV and remote SDR. Therefore, R2BM can be used as a monitoring method focusing on process control and risk identification at trial sites, as recommended by guidelines [5–7].

The R2BM method places an additional workload on the clinical trial staff unlike on-site monitoring. This additional workload for sponsors’ monitoring activities may trigger resistance from clinical trial staff [22]. The mean time to capture and upload photographs was approximately 26.5 min per visit per participant, which is longer than that (approximately 10 min) in our previous study [17]. The reason for this is that the type and number of source documents increased compared to those in our previous study, as the present study had many evaluations related to ophthalmic examinations recorded over several pages of EHR. In contrast, the R2BM method can eliminate the burden of on-site monitoring, such as booking a room, ensuring access to source documents for the requested participants, and ensuring sufficient time for clinical trial staff to support on-site monitoring. Therefore, it is necessary to discuss and identify in advance what source documents should be captured and to minimize the number of photographs captured for R2BM. This can avoid excessive additional workloads of R2BM compared to that of on-site monitoring reduced by R2BM. In addition, paying incentives for the additional workload of R2BM, with part of the travel cost of on-site monitoring saved by R2BM, would be one of the measures to reduce the resistance of clinical trial sites for R2BM.

An important factor for the success of R2BM is that clinical trial staff appropriately capture photographs of source documents. In this and our previous study [17], inappropriate photographs classified (A) to (F) were nominal. Almost all inappropriate photographs were recognized during R2BM, except for one photograph classified as category D (missed and undetected photos). The missed photograph contained information regarding an unscheduled visit. Therefore, it is crucial to discuss in advance which information must be captured and to communicate with clinical trial staff during the study on unexpected events such as unscheduled visits.

Prompt data entry and timely monitoring of clinical trials are essential for the early detection of issues and prevention of potential risks, which will aid in cost savings [23]. The median number of days from each participant visit to data entry in the eCRF was 1 day, indicating that data entry was performed on time. However, the median number of days from each participant’s visit to photograph upload was 80. This was more delayed than in the R2BM plan. Because on-site monitoring had already been conducted in the selected clinical trial when this study started, the clinical trial staff found supporting R2BM only for the present study burdensome and could not do it effectively because of their busy daily work. Therefore, it is necessary to evaluate R2BM compliance after establishing an appropriate R2BM implementation system with the clinical trial staff at the beginning of the trial.

DDC reduces the burden on data collection and transcription errors [16, 24]. The present study evaluated the impact of introducing DDC on the effectiveness of R2BM, assuming that DDC was introduced for data collection instead of EDC. In the complete dataset, 61.6% of the data did not require transcriptions and SDV by introducing DDC (category I), indicating that approximately 60% of the data collection burden and transcription errors would be avoided. In the R2BM plan, only critical data were planned to be verified; therefore, it would be sufficient in the R2BM methodology to conduct SDV to only the critical data classified as category II, which corresponds to 8.1% of the complete dataset. These findings indicate that the R2BM methodology combined with DDC can minimize source data requiring SDV and remotely verify them without any on-site visits.

Assuming that DDC was introduced, 35.8% of data errors in the critical data were classified as category I, indicating that these transcription errors would not occur or could be detected by data review without SDV. In addition, by combining DDC with data review, the proportion of data errors detectable only by SDV (category II-B) would be only 2.1% of the critical data. These findings suggest that the critical data errors detectable only by SDV were minimized. Therefore, with the R2BM methodology combined with DDC, the resources can be concentrated on SDR to detect critical process errors leading to systematic errors instead of on SDV to detect a small number of data errors. It would be beneficial for researchers to manage the quality of clinical trials using limited resources.

Furthermore, the R2BM methodology combined with DDC greatly benefits the clinical trial staff who capture photographs of source documents for R2BM. The present study showed that 45.0% of the uploaded photographs would not have to be captured by introducing DDC. The implementation of R2BM was delayed in this study; however, the combination of R2BM and DDC can potentially reduce the burden on clinical trial staff and facilitate the timely conduct of R2BM.

## Limitations

This prospective study included only eight participants from one clinical trial site. Therefore, it is necessary to evaluate the effectiveness of R2BM in various clinical trials with many participants from multiple centers. In addition, R2BM was started in the middle of the selected trial, where on-site monitoring had already been conducted. The quality of the clinical trial was already controlled by on-site monitoring to a certain extent when R2BM was started. Therefore, it is necessary to verify the effectiveness of quality control in the early phases of clinical trials by conducting R2BM from the start. The effectiveness of the combination of R2BM and DDC found in this study is only theoretical. Further evaluation of the synergistic effects of R2BM and DDC, including time and cost savings, is necessary.

## Conclusions

This study was conducted to determine the prospective effectiveness of R2BM. Although the study was conducted with eight participants at one clinical trial site, we found that R2BM detected 100% of critical data and process errors without on-site monitoring. Moreover, DDC can potentially reduce the burden of R2BM on the clinical trial staff, further improving the effectiveness of clinical trial monitoring using the R2BM methodology.

### Supplementary Information

Additional file 1: Table S1. Number of uploaded photographs and the time to capture and upload photographs. Table S2. Number (proportion) of uploaded photographs that can be reduced using direct data capture.

## Data Availability

The datasets generated and analyzed during the present study are not publicly available as the authors do not have permission to share these data.

## Abbreviations

CI: Confidence intervals
CRF: Case report form
DDC: Direct data capture
DM: Data manager
eCRF: Electronic case report form
EDC: Electronic data capture
EHR: Electronic health records
eSource Data: Electronic source data
IQR: Interquartile range
On-site CRAs: On-site monitoring clinical research associates
R2BM: Remote risk-based monitoring
RBM: Risk-based monitoring
Remote CRA: Remote monitoring clinical research associate
SD: Standard deviation
SDR: Source data review
SDV: Source data verification
