# Supplementary material for: Effectiveness of remote risk-based monitoring and potential benefits for combination with direct data capture
Source: Trials. 2024 Jun 14;25:384. doi: 10.1186/s13063-024-08242-2 (PMC11179298; doi:10.1186/s13063-024-08242-2)
Supplement: Supplementary file 1 — Additional file 1: Table S1. Number of uploaded photographs and the time to capture and upload photographs. Table S2. Number (proportion) of uploaded photographs that can be reduced using direct data capture. [file 13063_2024_8242_MOESM1_ESM.docx]

**Table S1. Number of uploaded photographs and the time to capture and upload photographs**

| (a) The number of uploaded photographs | | | | | | | | | | |
| --- | --- | --- | --- | --- | --- | --- | --- | --- | --- | --- |
|  | Participant No. | | | | | | | | Ground  total | Mean/visit  (SD) |
|  | 01 | 02 | 03 | 04 | 05 | 06 | 07 | 08 |  |  |
| Week 0 | 76 | 46 | 45 | 29 | 46 | 28 | 30 | 35 |  |  |
| Week 1 | 29 | - | 30 | 40 | 29 | 40 | 39 | 28 |  |  |
| Week 2 | 21 | - | 30 | 20 | 24 | 19 | 17 | 16 |  |  |
| Week 4 | 36 | - | 28 | 37 | 39 | 34 | 27 | 33 |  |  |
| Weeks 8 and 12 | 42 | - | 33 | 40 | 63 | 43 | 41 | 24 |  |  |
| Discontinuation | - | - | - | - | - | - | - | - |  |  |
| Total | 204 | 46 | 166 | 166 | 201 | 164 | 154 | 136 | 1237 | 34.4 (11.9) |
| (b) Time (min) to capture and upload photographs | | | | | | | | | | |
|  | Participant No. | | | | | | | | Ground  total | Mean/visit  (SD) |
|  | 01 | 02 | 03 | 04 | 05 | 06 | 07 | 08 |  |  |
| Week 0 | 80 | 40 | 40 | 40 | 40 | 40 | 40 | 40 |  |  |
| Week 1 | 20 |  | 20 | 20 | 20 | 20 | 20 | 20 |  |  |
| Week 2 | 20 | - | 20 | 20 | 20 | 20 | 20 | 20 |  |  |
| Week 4 | 20 | - | 20 | 20 | 20 | 20 | 20 | 20 |  |  |
| Weeks 8 and 12 | 25 | - | 25 | 25 | 25 | 25 | 25 | 25 |  |  |
| Discontinuation | - | - | - | - | - | - | - | - |  |  |
| Total | 165 | 40 | 125 | 125 | 125 | 125 | 125 | 125 | 955 | 26.5 (11.8) |

Participant No. 2 was discontinued after the initiation of treatment due to a serious adverse event.

**Table S2. Number (proportion) of uploaded photographs that can be reduced using direct data capture**

|  | Participant No. | | | | | | | | Ground  total |
| --- | --- | --- | --- | --- | --- | --- | --- | --- | --- |
|  | 01 | 02 | 03 | 04 | 05 | 06 | 07 | 08 |  |
| Week 0 | 14/76  (18.4%) | 26/46  (56.5%) | 16/45  (35.6%) | 14/29  (48.3%) | 17/46  (37.0%) | 13/28  (46.4%) | 11/30  (36.7%) | 13/35  (37.1%) |  |
| Week 1 | 18/29  (62.1%) | - | 19/30  (63.3%) | 28/40  (70.0%) | 18/29  (62.1%) | 14/40  (35.0%) | 18/39  (46.2%) | 11/28  (39.3%) |  |
| Week 2 | 11/21  (52.4%) | - | 12/30  (40.0%) | 12/20  (60.0%) | 12/24  (50.0%) | 10/19  (52.6%) | 10/17  (58.8%) | 9/16  (56.2%) |  |
| Week 4 | 16/36  (44.4%) | - | 19/28  (67.9%) | 17/37  (45.9%) | 16/39  (59.0%) | 18/34  (52.9%) | 10/27  (37.0%) | 14/33  (42.4%) |  |
| Weeks 8 and 12 | 19/42  (45.2%) | - | 17/33  (51.5%) | 17/40  (42.5%) | 30/63  (47.6%) | 14/43  (32.6%) | 20/41  (48.8%) | 4/24  (16.7%) |  |
| Discontinuation | - | - | - | - | - | - | - | - |  |
| Total | 78/204  (38.2%) | 26/46  (56.5%) | 83/166  (50.0%) | 88/166  (53.0%) | 93/201  (46.3%) | 69/164  (42.1%) | 69/154  (44.8%) | 51/136  (37.5%) | 557/1237  (45.0%) |

Participant No. 2 was discontinued after the initiation of treatment due to a serious adverse event.
